# Effects of Statins on the Incidence and Mortality of Sepsis in Patients with New Cancer Diagnosis

**DOI:** 10.3390/jcm10153427

**Published:** 2021-07-31

**Authors:** Andry Van de Louw, Austin Cohrs, Douglas Leslie

**Affiliations:** 1 Division of Pulmonary and Critical Care Medicine, Penn State Health Milton S Hershey Medical Center, Hershey, PA 17033, USA; 2 Department of Public Health Sciences, Penn State Health Milton S Hershey Medical Center, Hershey, PA 17033, USA; acohrs@pennstatehealth.psu.edu (A.C.); Dleslie@phs.psu.edu (D.L.)

**Keywords:** statin, sepsis, cancer

## Abstract

Statins have been associated with improved survival in cancer patients and with decreased incidence and mortality of sepsis in different populations. Our objective was to assess whether newly diagnosed cancer patients on statins had decreased incidence and mortality of sepsis. We analyzed a US database and included 119,379 patients with a new cancer diagnosis (age 55 (50–60) years, 61% female), 19,468 of them (16%) receiving statins. Statins users were older and presented more comorbidities. After adjustment for baseline characteristics, statin use was associated with decreased death hazard (HR 0.897, 95% CI 0.851–0.945, *p* < 0.0001). The cumulative incidence of sepsis reached 10% at 5 years but statin use was not significantly associated with sepsis hazard (subdistribution hazard ratio 0.990, 95% CI 0.932–1.050, *p* = 0.73), including in sensitivity analyzes in patients with hematological malignancy or sepsis within 1 year. In patients subsequently hospitalized with sepsis, hospital mortality was 23% and statin use was not associated with mortality (odds ratio 0.952, 95% CI 0.829–1.091, *p* = 0.48), including in sensitivity analyzes in patients with septic shock and use of statins at the time of sepsis. In summary, treatment with statin at the time of new cancer diagnosis is not associated with a decreased incidence and mortality of sepsis.

## 1. Introduction

Statins are HMG-CoA reductase inhibitors and are mostly used to reduce blood cholesterol levels in patients with cardiovascular risk. However, increasing evidence suggests that their effects go beyond decreasing blood cholesterol and reducing cardiovascular mortality [1].

Data from cancer registries [2] and meta-analyses of large cohort studies [3] suggest that statin use in patients with cancer is associated with decreased mortality. Two main mechanisms have been proposed to account for this observed association: (1) statins inhibit tumor growth and induce apoptosis in a number of tumor types in vitro [4,5] and their anti-carcinogenic activity has been demonstrated in animal models of solid and hematological cancers [6,7], (2) statins have been shown to decrease cardiovascular mortality in high- and intermediate-cardiovascular risk patients [8,9]. However, statins also have pleiotropic properties which might be beneficial during sepsis: they have anti-inflammatory, anticoagulant and anti-oxidative activity [10] as well as direct antimicrobial activity against certain organisms [11].

Septic shock is a leading cause for Intensive Care Unit (ICU) admission in patients with cancer (42% of patients in [12]) and is associated with high hospital mortality (56% in [13]). The impact of statin use on mortality in sepsis has been investigated in unselected ICU patients with inconsistent conclusions: while observational and data registry studies suggest that treatment with statins in atherosclerotic patients decrease the incidence of subsequent sepsis [14] and improves sepsis 30-day survival [15], randomized control trials have not shown an impact of post-admission statin administration on sepsis mortality [16].

Whether chronic statin administration decreases the risk and mortality of sepsis in cancer patients is a significant gap in the current knowledge; if this hypothesis was confirmed, it might provide an explanatory mechanism for the improved overall survival reported in cancer patients receiving statins, and could lead to therapeutic interventions in selected patients at risk of developing infections (chemotherapy-induced neutropenia, high doses steroids, hematopoietic stem cell transplant).

The objective of this study was to ascertain whether chronic statin treatment is associated with a decreased incidence and mortality of sepsis in patients with new cancer diagnosis.

## 2. Methods

This retrospective registry study was approved by the Pennsylvania State University institutional review board and used 2005–2014 data from the Truven Health MarketScan database. The database is a commercially available health insurance claims database. It includes claims data for a sample of more than 245 million privately insured people in all 50 US states, including demographic characteristics, health care utilization and costs, dates of service, diagnosis codes and procedure codes. The data represent claims from clinicians, hospitals, and pharmacies that have been adjudicated for payment and are obtained directly from a convenience sample of large employers and health plans that agree to participate in the database. Marketscan does not include patients on Medicare (≥65-year-old). Truven Health has a quality-control process to verify that the data meet criteria for quality and completeness. This database has been used in multiple other studies [17,18], including studies examining complications and follow-up care after health care procedures [18].

All patients in the database who met the following criteria were included: (1) age ≥ 40 years (as statin use is rarer in younger patients); (2) diagnosis of cancer between 2006 and 2014 based on ICD-9 codes: 140–149.9 for head and neck, 150–159.9 for gastrointestinal system, 160–165.9 for respiratory system, 170–176.9 for musculoskeletal and breast cancers, 179–189.9 for genitourinary system, 190–199.9 for other and unspecified sites, 200–209.9 for hematological malignancies, 235–238.9 for cancers of uncertain behavior, 239–239.9 for cancers of unspecified nature; (3) to include only patients with new cancer diagnoses, patients had to be continuously enrolled in the database, without a diagnosis code for cancer or a procedure code for chemotherapy or radiotherapy, for at least 12 months prior to the index date of cancer diagnosis; (4) administration of chemotherapy within 6 months of the index date of cancer diagnosis, based on either one of ICD-9 CM codes of 99.25, V58.1x, V66.2, V67.2, CPT-4 codes of 96400–96549, J9000–J9999, Q0083–Q0085, revenue center codes of 0331, 0332, and 0335; (5) to ensure a minimal follow-up, patients had to be continuously enrolled in the database at least 12 months after the index date of cancer diagnosis, unless they died or were admitted on hospice.

Patients were defined as statin users if they had ≥1 prescription for ≥30 days filled within 4 months prior to index date of cancer diagnosis for any of the following drugs: atorvastatin, fluvastatin, lovastatin, pravastatin, rosuvastatin, simvastatin or pitavastatin.

All included patients, statin users or not, were screened for hospital admissions with ICD-9 diagnoses of sepsis (995.91), severe sepsis (995.92) or septic shock (785.52). For these admissions, ICD-9 principal and secondary diagnoses (up to 15 per admission), procedures codes (up to 15 per admission) and discharge status were collected. A modified Charlson comorbidity index, not taking into account the diagnoses of malignancy, was used to assess comorbidities based on diagnoses present in the database within 1 year prior to cancer diagnosis. Four groups were defined based on the modified scores: 0, 1–2, 3–4 and ≥5.

In order to assess survival status over time, we screened follow-up information available in the database and used discharge status for the last inpatient admission (regardless of diagnoses of sepsis) as well as physician office visits and outpatient prescription fillings (whichever the latest).

### Statistical Analysis

Continuous variables were described as medians (interquartile ranges (IQRs)) and categorical variables as numbers (percentages). Groups were compared using Fisher`s exact test for categorical variables and Wilcoxon rank-sum test for continuous variables. The effect of statin use on overall survival was assessed using a Cox proportional hazard model also including age, gender, cancer site and modified Charlson comorbidity index group as covariates. Hazard proportionality, linearity for the covariate age and outliers were checked. A sensitivity analysis was performed including only patients with solid tumors and adding presence of metastases at diagnosis to the model. To assess the cumulative incidence of sepsis, we used a competing risk analysis taking into account death without sepsis as a competing event for sepsis. Incidences of sepsis and death without sepsis were computed using the ‘comprsk’ package and a Fine and Gray model was used to ascertain the effect of covariates on subdistribution hazard ratios (SHR) for sepsis. The proportionality of SHR was carefully checked by visual inspection of the plots of residuals. Sensitivity analyses were performed, restricting analysis to patients with hematological malignancy or sepsis within the first year of cancer diagnosis. To assess the effect of statin use on hospital mortality for the subset of patients who developed sepsis, variables associated with mortality in univariate analysis with *p* < 0.1 were entered in a backward stepwise logistic regression model and statin use was forced into the model. Multicollinearity, linearity for continuous variables and outliers were carefully checked.

Analyses were performed using R 3.3.2 (http://www.R-project.org/, accessed on 15 January 2021) and *p* < 0.05 was considered for statistical significance.

## 3. Results

The cohort included 119,379 patients (age 55 (50–60) years, 61% female) and has been already described in a previous publication in detail [19]. The follow-up time for the cohort was 881 (511–1566) days. There were 19,468 statin users (16.3%) and 99,911 non-statin users (83.7%): statin users were older, more frequently male and with cardiovascular, pulmonary or renal comorbidities (Table 1). As a result, statin users more frequently had low (1–2), moderate (3–4) or high (≥5) modified Charlson comorbidity index while more non statin users had a score of 0 (Table 1). Statin users more frequently had head and neck, lung or genitourinary cancer whereas musculoskeletal and breast cancers were more frequent in non-statin users.

### 3.1. Effect of Statin Use on Overall Survival

Table 2 summarizes the results of the Cox model assessing the effect of covariates on overall survival. The following variables were associated with increased hazard for death: male gender, age and modified Charlson comorbidity index. As compared to hematological malignancy, death hazards were higher for gastrointestinal or lung cancer and lower for genitourinary, head and neck and musculoskeletal/breast cancers. Statin use was associated with a decreased death hazard (HR 0.897, 95% CI 0.851–0.945, *p* < 0.0001). Similar results were obtained in the sensitivity analysis including only patients with solid tumors (HR for statin use 0.863, 95% CI 0.816–0.913, *p* < 0.0001) with the addition of metastases at diagnosis being significantly associated with mortality (HR 2.286, 95% CI 2.173–2.404, *p* < 0.0001).

### 3.2. Effect of Statin Use on the Incidence of Sepsis

**Figure 1:**
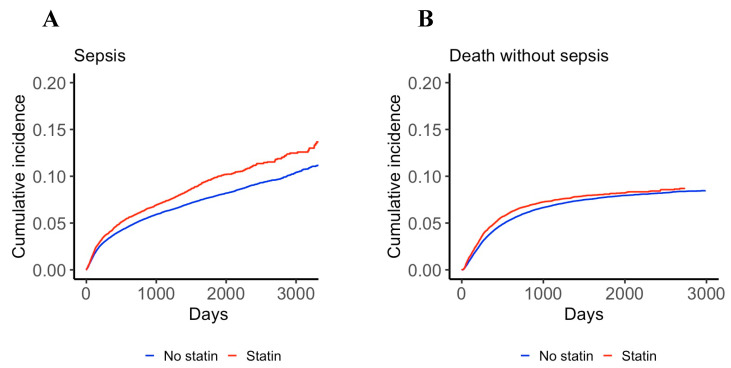
Cumulative incidence of sepsis (panel **A**) and death without sepsis (treated as a competing event, panel **B**) in patients with a new cancer diagnosis receiving or not receiving statins prior to cancer diagnosis.

Figure 1 displays the cumulative incidence of sepsis and death without sepsis over time: the incidence of sepsis was significantly higher in statin users (*p* < 0.0001) and reached about 10% after 5 years. Although the incidence of death without sepsis was also statistically higher in statin users (*p* = 0.003), the curves appeared much closer between the 2 groups compared to the sepsis incidence curves.

Table 3 reports the results of the Fine and Gray model: when adjusting for covariates, statin use was not significantly associated with hazard of sepsis (subdistribution hazard ratio 0.990, 95% CI 0.932–1.050, *p* = 0.73), whereas male gender, age and modified Charlson comorbidity index group all increased the hazard of sepsis. As compared to hematological malignancy, all other cancer sites were associated with decreased sepsis hazard. Statin use was not associated with sepsis hazard in sensitivity analyses restricted to patients with hematological malignancy (SHR 1.083, 95% CI 0.938–1.250, *p* = 0.28) and to patients with sepsis within 1 year of cancer diagnosis (SHR 0.926, 95% CI 0.855–1.002, *p* = 0.06).

### 3.3. Effect of Statin Use on Sepsis Mortality

Overall, 7743 patients were hospitalized with sepsis after 289 (106–721) days, 2194 of them (28.3%) developed septic shock. Discharge status was available for 7334 patients, the hospital mortality was 23% (*n* = 1704) overall and was not different between statin users (*n* = 1365) and non-users (22.7% versus 23.3%, *p* = 0.51), although statin users more often developed septic shock (31.5% versus 27.6%, *p* = 0.03). Hospital mortality was 34% (486 out of 1429 patients) for the subset of patients with severe sepsis and 44% (919 out of 2087 patients) for those with septic shock, without significant difference between statin users and non-users (33.8% versus 34.0%, *p* = 0.94 and 41.0% versus 44.8%, *p* = 0.15, respectively). Among statin users, the last recorded prescription of statin was 57 (22–158) days prior to admission with sepsis. In univariate analysis, hospital survivors were younger, more frequently females, and although the distribution of the modified Charlson comorbidity score was overall not significantly different from non survivors, they had less frequently peripheral vascular disease, cerebrovascular disease and chronic pulmonary disease (Table 4). Survivors had more musculoskeletal, breast and genitourinary cancers, less lung cancers, and less metastases across the board. Non-survivors more frequently developed severe sepsis and septic shock, but the prevalence of statin use was not different between survivors and deceased patients. In multivariate analysis (Table 5), statin use was not associated with hospital mortality (OR 0.952, 95% CI 0.829–1.091, *p* = 0.48), whereas age, history of cerebrovascular disease and metastases were associated with increased mortality. As compared to hematological malignancy, musculoskeletal or breast cancer and genitourinary cancer were associated with decreased mortality (Table 5). Statin use was not associated with mortality in a sensitivity analysis restricted to the 2194 patients who developed septic shock (OR 0.823, 95% CI 0.657–1.029, *p* = 0.09), or in another sensitivity analysis restricted to the 450 statin users whose last recorded prescription was within 30 days of admission with sepsis (OR 0.914, 95% CI 0.729–1.141, *p* = 0.43).

## 4. Discussion

In this analysis of a large database of US adults aged 40 years and older and with a new cancer diagnosis, our main findings were that statin use prior to cancer diagnosis was associated with a decreased hazard for death but not with a decreased incidence of sepsis or sepsis-related mortality. The crude incidence of sepsis was higher in statin users but after adjustment for age, gender, comorbidities and type of cancer no difference was observed compared to non-statin users. Statin use was not associated with hospital mortality in the subset of cancer patients who developed sepsis.

The association between statin use and decreased mortality in cancer patients had been already reported: in a Danish population-based study including approximately 296,000 patients with cancer, regular use of statin before cancer diagnosis was associated with decreased hazard for death from any cause (HR 0.85, 95% CI 0.83–0.87) and death from cancer (HR 0.85, 95% CI 0.82–0.87) [2]. Studies focused on specific types of cancer have suggested similar benefits in patients with pancreatic [20], breast [21], gynecologic [22], kidney [23] or colorectal [24] tumors, and a meta-analysis of 55 studies also concluded to a decreased risk of mortality in statin users (HR 0.70, 95% 0.66–0.74) [3]. The effect size observed in our population was smaller as compared to these studies but remained significant (HR 0.90, 95% CI 0.85–0.95).

Several anti-tumoral effects of statins have been proposed to account for this beneficial effect: by inhibiting HMG-CoA reductase, statins alter the metabolism of cholesterol, a major component of cell membrane, and also decrease the synthesis of mevalonate, a precursor of products regulating the cell cycle [25], resulting in inhibition of tumor cell growth. Statins promote apoptosis by upregulating pro-apoptotic proteins while downregulating anti-apoptotic ones (bcl-2) [25], and also impair the metastatic potential of tumor cells by inhibiting cell migration, attachment to the extracellular matrix and invasion of the basement membrane [25]. Depending on drug concentration and tumor cell type, statins can also display anti-angiogenic properties (VEGF downregulation) [26].

As cancer patients are at higher risk of dying from cardiovascular disease than the general population [27], the proven decrease in cardiovascular mortality associated with statins in high cardiovascular risk patients [8,9] may be driving the improved overall survival observed in statin users cancer patients [2,3].

However, statins have additional properties (anti-inflammatory, immunomodulatory), independent of their lipid-lowering ability, which could also account for their beneficial effect in cancer patients. Specifically, statins gained attention after early reports of reduced mortality in murine models of sepsis [28,29] and improved survival in patients with sepsis in meta-analysis of mostly observational studies [30]. However, randomized control trials published later [31,32,33] and recent meta-analyses including only randomized control trials [16,34] concluded to the lack of effect of statins on mortality. If there is no convincing evidence that statins as a treatment initiated for sepsis improves survival, the effect of chronic statin use on the incidence and outcome of sepsis is less clear, as most randomized control trials excluded patients previously on statins [31,32]. Several studies have suggested that chronic statin use may be associated with decreased rate of severe sepsis, ICU admission [35] and mortality [36,37]. A large Canadian population-based cohort analysis including approximately 141,000 patients reported that the use of statins in patients with atherosclerosis was associated with a reduced risk of subsequent sepsis (HR 0.81, 95% CI 0.72–0.90), severe sepsis (HR 0.83, 95% CI 0.70–0.97) and fatal sepsis (0.75, 95% CI 0.61–0.93) after adjustment for demographic characteristics, comorbidities and sepsis risk factors [14]. Similar results were reported in patients with end stage renal disease on dialysis [38]. Whether chronic statin use is similarly associated with reduced incidence and mortality of sepsis in cancer patients and whether this could be driving the improved overall survival in statin users has not been investigated. This is relevant as cancer patients have higher incidence rates for sepsis than non-cancer patients (relative risk of 9.77, 95% CI 9.67–9.88 in [39]) with an associated mortality of approximately 37% for severe sepsis [40]. In the present study, including a large population of patients with new cancer diagnosis, we confirmed that prior statin use was associated with a decreased death hazard overall, but we did not observe an effect of statin use on the incidence of sepsis or sepsis-related mortality, even in multiple sensitivity analyses. The incidence of sepsis was about 5% at 1 year and 10% at 5 years in our population, in agreement with a large Australian population-based study reporting a 1-year incidence of 6.4% in cancer patients [41]. Statin users had a higher crude incidence of sepsis, but they also had different baseline characteristics as compared with non statin users and after adjustment for these characteristics the effect of statin use on sepsis incidence was no longer observed. Hospital mortality was 23% in our patients with sepsis and 34% in those with severe sepsis, in agreement with previous studies in cancer patients with sepsis [40,42].

This study has several limitations: the first one, in common with most population-based observational studies discussed above, is the possibility of a selection bias. We cannot rule out a « healthy user » effect, because socioeconomically more privileged patients may be more likely to receive preventive treatments like statins and also more likely to have regular medical follow-up and healthy lifestyle [43]. The comparison of non-users with prevalent users (who started statins before cancer diagnosis) is also subject to selection bias [44] and a recent study trying to overcome this possible bias actually did not conclude to an effect of statins initiated after cancer diagnosis on cancer-related or all-cause mortality [45]. However, inclusion of incident users only (who started statins after cancer diagnosis) would have been problematic in our study, as sepsis may occur early after cancer diagnosis, especially in patients receiving chemotherapy, and the time required for statins to exert their full anti-inflammatory effect, among others, is unknown but may amount to several weeks [46,47]. Second, we did not adjust our analyses for the dose, duration of administration and specific drug used, whereas some studies have suggested that the effects of statin may vary with drugs [15] and dosage [48]. Finally, although the analyses were adjusted for baseline characteristics including comorbidities, there is always the possibility of confounding factors that remained unaccounted for.

## 5. Conclusions

In summary, in a large population-based study of patients 40 years and older with a new cancer diagnosis, we observed that statin use prior to diagnosis was associated with a decreased hazard for death, but that the incidence and mortality of sepsis did not differ between statin users and non-users. Further studies are warranted to definitely confirm the effects of statins on survival in cancer patients and to understand the mechanisms of their potential benefit.

## Figures and Tables

**Table 1:** Characteristics of the population and comparison of patients according to statin administration.

	No Statin (*n* = 99,911)	Statin (*n* = 19,468)	Total (*n* = 119,379)	*p* Value
**Female, *n* (%)**	63,174 (63.2%)	9758 (50.1%)	72,932 (61.1%)	<0.001
**Age, years**	54 (49–59)	58 (54–61)	55 (50–60)	<0.001
**Comorbidities:**				
***Myocardial infarction, n (%)***	478 (0.5%)	459 (2.4%)	937 (0.8%)	<0.001
***Congestive heart failure, n (%)***	1126 (1.1%)	539 (2.8%)	1665 (1.4%)	<0.001
***Peripheral vascular disease, n (%)***	2010 (2.0%)	990 (5.1%)	3000 (2.5%)	<0.001
***Cerebrovascular disease, n (%)***	2212 (2.2%)	1091 (5.6%)	3303 (2.8%)	<0.001
***Chronic pulmonary disease, n (%)***	10,222 (10.2%)	2462 (12.6%)	12,684 (10.6%)	<0.001
***Renal disease, n (%)***	1265 (1.3%)	574 (2.9%)	1839 (1.5%)	<0.001
**Modified Charlson comorbidity index risk, *n* (%)**				<0.001
mCCI = 0	68,540 (70.5%)	9454 (48.7%)	77,994 (66.9%)	
mCCI 1–2	25,773 (26.5%)	8605 (44.3%)	34,378 (29.5%)	
mCCI 3–4	2162 (2.2%)	1121 (5.8%)	3283 (2.8%)	
mCCI ≥ 5	723 (0.7%)	226 (1.2%)	949 (0.8%)	
**Cancer site:**				
***Head and neck, n (%)***	1771 (1.8%)	407 (2.1%)	2178 (1.8%)	0.002
***Gastrointestinal, n (%)***	15,728 (15.7%)	3132 (16.1%)	18,860 (15.8%)	0.226
***Lung, n (%)***	7504 (7.5%)	1918 (9.9%)	9422 (7.9%)	<0.001
***Musculoskeletal and breast, n (%)***	32,556 (32.6%)	4773 (24.5%)	37,329 (31.3%)	<0.001
***Genitourinary, n (%)***	13,404 (13.4%)	3337 (17.1%)	16,741 (14.0%)	<0.001
***Hematological malignancy, n (%)***	9160 (9.2%)	1790 (9.2%)	10,950 (9.2%)	0.907
**Metastases, *n* (%)**	10,755 (10.8%)	2027 (10.4%)	12,782 (10.7%)	0.145

**Table 2:** Summary of the Cox proportional hazard model assessing the effect of covariates on the overall survival.

Covariate	Hazard Ratio	95% Confidence Interval	*p*
**Female gender**	0.857	0.822–0.894	<0.0001
**Age**	1.023	1.019–1.026	<0.0001
**Modified Charlson comorbidity index group** **(0 as the reference):**			
***mCCI 1–2***	1.392	1.334–1.452	<0.0001
***mCCI 3–4***	1.874	1.711–2.052	<0.0001
***mCCI ≥ 5***	2.233	1.918–2.560	<0.0001
**Cancer site (hematological malignancy as the reference):**			
***Gastrointestinal***	1.160	1.082–1.244	<0.0001
***Genitourinary***	0.405	0.370–0.443	<0.0001
***Head and neck***	0.522	0.435–0.626	<0.0001
***Musculoskeletal and breast***	0.273	0.250–0.297	<0.0001
***Lung***	2.262	2.105–2.432	<0.0001
**Statin use**	0.897	0.851–0.945	<0.0001

Hazard ratios for death were derived from the multivariate Cox proportional hazard model including all covariates above. mCCI: modified Charlson comorbidity index.

**Table 3:** Summary of the Fine and Gray model assessing the effect of covariates on the hazard of sepsis, treating death without sepsis as a competing event.

Covariate	Subdistribution Hazard Ratio	95% Confidence Interval	*p*
**Female gender**	0.931	0.885–0.979	0.005
**Age**	1.017	1.013–1.021	<0.0001
**Modified Charlson comorbidity index group** **(0 as the reference):**			
***mCCI 1–2***	1.438	1.368–1.512	<0.0001
***mCCI 3–4***	2.243	2.028–2.482	<0.0001
***mCCI ≥ 5***	2.899	2.472–3.400	<0.0001
**Cancer site (hematological malignancy as the reference):**			
***Gastrointestinal***	0.879	0.817–0.946	0.0006
***Genitourinary***	0.445	0.407–0.486	<0.0001
***Head and neck***	0.580	0.486–0.692	<0.0001
***Musculoskeletal and breast***	0.265	0.243–0.290	<0.0001
***Lung***	0.771	0.703–0.845	<0.0001
**Statin use**	0.990	0.932–1.050	0.73

**Table 4:** Comparison between hospital survivors and non-survivors among cancer patients hospitalized with sepsis during follow-up.

	Survivors (*n* = 5630)	Non Survivors (*n* = 1704)	*p* Value
**Female, *n* (%)**	2926 (52.0%)	817 (47.9%)	0.012
**Age, years**	56 (50–59)	57 (52–60)	<0.001
**Comorbidities:**			
***Myocardial infarction, n (%)***	71 (1.3%)	13 (0.8%)	0.069
***Congestive heart failure, n (%)***	157 (2.8%)	51 (3.0%)	0.130
***Peripheral vascular disease, n (%)***	213 (3.8%)	77 (4.5%)	0.025
***Cerebrovascular disease, n (%)***	176 (3.1%)	83 (4.9%)	<0.001
***Chronic pulmonary disease, n (%)***	776 (13.8%)	260 (15.3%)	0.017
***Renal disease, n (%)***	216 (3.8%)	61 (3.6%)	0.056
**Modified Charlson comorbidity index risk, *n* (%)**			0.299
mCCI = 0	3033 (55.3%)	881 (53.6%)	
mCCI 1–2	2034 (37.1%)	613 (37.3%)	
mCCI 3–4	301 (5.5%)	108 (6.6%)	
mCCI ≥ 5	114 (2.1%)	42 (2.6%)	
**Statin use, *n* (%)**	1053 (18.7%)	312 (18.3%)	0.514
**Cancer site:**			
***Head and neck, n (%)***	111 (2.0%)	28 (1.6%)	0.420
***Gastrointestinal, n (%)***	1306 (23.2%)	399 (23.4%)	0.282
***Lung, n (%)***	505 (9.0%)	235 (13.8%)	<0.001
***Musculoskeletal and breast, n (%)***	900 (16.0%)	182 (10.7%)	<0.001
***Genitourinary, n (%)***	683 (12.1%)	152 (8.9%)	<0.001
***Hematological malignancy, n (%)***	921 (16.4%)	301 (17.7%)	0.436
**Metastases, *n* (%)**	775 (13.8%)	327 (19.2%)	<0.001
**Severe sepsis, *n* (%)**	943 (16.7%)	486 (28.5%)	<0.001
**Septic shock, *n* (%)**	1168 (20.7%)	919 (53.9%)	<0.001

**Table 5:** Summary of the multivariate logistic regression model assessing the effect of covariates on hospital mortality in the subset of cancer patients hospitalized with sepsis.

Covariate	Odds Ratio	95% Confidence Interval	*p*
**Statin use**	0.952	0.829–1.091	0.479
**Age**	1.018	1.008–1.028	<0.001
**History of cerebrovascular disease**	1.439	1.071–1.920	0.014
**History of myocardial infarction**	0.680	0.375–1.165	0.179
**Cancer site (hematological malignancy as the reference):**			
***Gastrointestinal***	0.914	0.763–1.095	0.330
***Genitourinary***	0.683	0.545–0.852	<0.001
***Head and neck***	0.629	0.385–0.993	0.055
***Musculoskeletal and breast***	0.694	0.564–0.853	<0.001
***Lung***	1.176	0.944–1.464	0.146
**Metastases**	1.300	1.106–1.526	0.001

## Data Availability

Restrictions apply to the availability of these data. Data was obtained from IBM Marketscan and are available from the authors with the permission of IBM Marketscan.
